# The Burden of Clostridioides Difficile Infection during the COVID-19 Pandemic: A Retrospective Case-Control Study in Italian Hospitals (CloVid)

**DOI:** 10.3390/jcm9123855

**Published:** 2020-11-27

**Authors:** Guido Granata, Alessandro Bartoloni, Mauro Codeluppi, Ilaria Contadini, Francesco Cristini, Massimo Fantoni, Alice Ferraresi, Chiara Fornabaio, Sara Grasselli, Filippo Lagi, Luca Masucci, Massimo Puoti, Alessandro Raimondi, Eleonora Taddei, Filippo Fabio Trapani, Pierluigi Viale, Stuart Johnson, Nicola Petrosillo, on behalf of the CloVid Study Group

**Affiliations:** 1 Clinical and Research Department for Infectious Diseases, Severe and Immunedepression-Associated Infections Unit, National Institute for Infectious Diseases L. Spallanzani IRCCS, 00149 Rome, Italy; nicola.petrosillo@inmi.it; 2 Department of Infectious Diseases, Careggi Hospital, University of Florence, 50121 Florence, Italy; alessandro.bartoloni@unifi.it (A.B.); filippo.lagi@unifi.it (F.L.); 3 Infectious Diseases Unit, Guglielmo da Saliceto Hospital, 29121 Piacenza, Italy; m.codeluppi@ausl.pc.it (M.C.); s.grasselli@ausl.pc.it (S.G.); 4 Infectious Diseases Unit, Rimini-Forlì-Cesena Hospitals, 48121 Rimini, Italy; ilaria.contadini@auslromagna.it (I.C.); francesco.cristini@auslromagna.it (F.C.); 5 Dipartimento di Scienze di Laboratorio e Infettivologiche —Fondazione Policlinico A. Gemelli IRCCS, Via della Pineta Sacchetti, 00168 Rome, Italy; massimo.fantoni@policlinicogemelli.it (M.F.); luca.masucci@policlinicogemelli.it (L.M.); 6 Infectious Diseases Unit, ASST Cremona, 26100 Cremona, Italy; alice.ferraresi@asst-cremona.it (A.F.); c.fornabaio@asst-cremona.it (C.F.); 7 Infectious Diseases Unit, ASST Grande Ospedale Metropolitano Niguarda, 20162 Milan, Italy; massimo.puoti@ospedaleniguarda.it (M.P.); alessandro.raimondi@ospedaleniguarda.it (A.R.); 8 Dipartimento di Sicurezza e Bioetica—Sezione di Malattie Infettive—Fondazione Policlinico A. Gemelli IRCCS, Via della Pineta Sacchetti, 00168 Rome, Italy; eleonora.taddei@policlinicogemelli.it; 9 Department of Medical and Surgical Sciences, Infectious Diseases Unit, Alma Mater Studiorum–University of Bologna, 40126 Bologna, Italy; filippofabio.trapani@aosp.bo.it (F.F.T.); pierluigi.viale@unibo.it (P.V.); 10 Research Service, Hines VA Hospital and Infectious Disease Section, Loyola University Medical Center, Maywood, IL 60153, USA; stuart.johnson2@va.gov

**Keywords:** *Clostridioides difficile* infection, coronavirus disease 2019 pandemic, risk factors, hospital-onset, case-control study

## Abstract

Data on the burden of *Clostridioides difficile* infection (CDI) in Coronavirus Disease 2019 (COVID-19) patients are scant. We conducted an observational, retrospective, multicenter, 1:3 case (COVID-19 patients with CDI)-control (COVID-19 patients without CDI) study in Italy to assess incidence and outcomes, and to identify risk factors for CDI in COVID-19 patients. From February through July 2020, 8402 COVID-19 patients were admitted to eight Italian hospitals; 38 CDI cases were identified, including 32 hospital-onset-CDI (HO-CDI) and 6 community-onset, healthcare-associated-CDI (CO-HCA-CDI). HO-CDI incidence was 4.4 × 10,000 patient-days. The percentage of cases recovering without complications at discharge (i.e., pressure ulcers, chronic heart decompensation) was lower than among controls (*p* = 0.01); in-hospital stays was longer among cases, 35.0 versus 19.4 days (*p* = 0.0007). The presence of a previous hospitalisation (*p* = 0.001), previous steroid administration (*p* = 0.008) and the administration of antibiotics during the stay (*p* = 0.004) were risk factors associated with CDI. In conclusions, CDI complicates COVID-19, mainly in patients with co-morbidities and previous healthcare exposures. Its association with antibiotic usage and hospital acquired bacterial infections should lead to strengthen antimicrobial stewardship programmes and infection prevention and control activities.

## 1. Introduction

Since 31 December 2019, when the World Health Organization (WHO) was informed of an outbreak of respiratory disease affecting the city of Wuhan, the world has been shaken by the most profound health crisis of the last several decades [1,2]. Coronavirus Disease 2019 (COVID-19), caused by the severe acute respiratory syndrome coronavirus 2 (SARS-CoV-2), has spread rapidly worldwide with the consequence of causing a serious health threat to humans on every continent. At present, more than thirty million people are known to have been infected, which has placed a great burden on health care systems and heightened anxiety and psychological stress of medical staff [3]. The lack of high-level evidence, inherent to the novelty and rapid spread of COVID-19, has led to the adoption of heterogeneous therapeutic management approaches, often without a clear distinction between evidence-based data and expert opinion in informing treatment choices. The high number of hospitalizations, shortage of beds, especially in critical areas, and the need for healthcare worker protection have challenged compliance with infection control and antibiotic stewardship programs in most health-care facilities facing this emergent threat of COVID-19 [4]. During the pandemic many health-care facilities gave priority to the protection of their healthcare workers from COVID-19, reducing attention to the prevention of other bacterial infections transmitted by interpersonal contact. Moreover, most of the early recommendations for the management of COVID-19 patients considered the use of empirical antibiotic treatment, resulting in large usage of antimicrobials in COVID-19 patients. Up to 94% of COVID-19 patients have been reported to receive empirical antibiotic therapy during their hospital stay [4,5,6,7,8,9]. Bacterial superinfections have been described in the course of COVID-19 disease and early reports of *Clostridioides difficile* infection (CDI) co-infection have been published [10,11]. CDI is commonly associated with the use of broad-spectrum antibiotics, absence of antimicrobial stewardship, inadequate infection control and hospital overcrowding [12]. Currently, we do not have a clear picture of the burden of CDI in COVID-19 patients and there is a lack of data on the prevalence and clinical manifestations of CDI in COVID-19 patients.

The aim of this study was to assess the incidence of CDI in hospitalized COVID-19 patients, to describe the clinical characteristics and outcomes of COVID-19 patients with CDI and to identify risk factors for the onset of CDI in COVID-19 patients.

## 2. Materials and Methods

**Figure 1:**
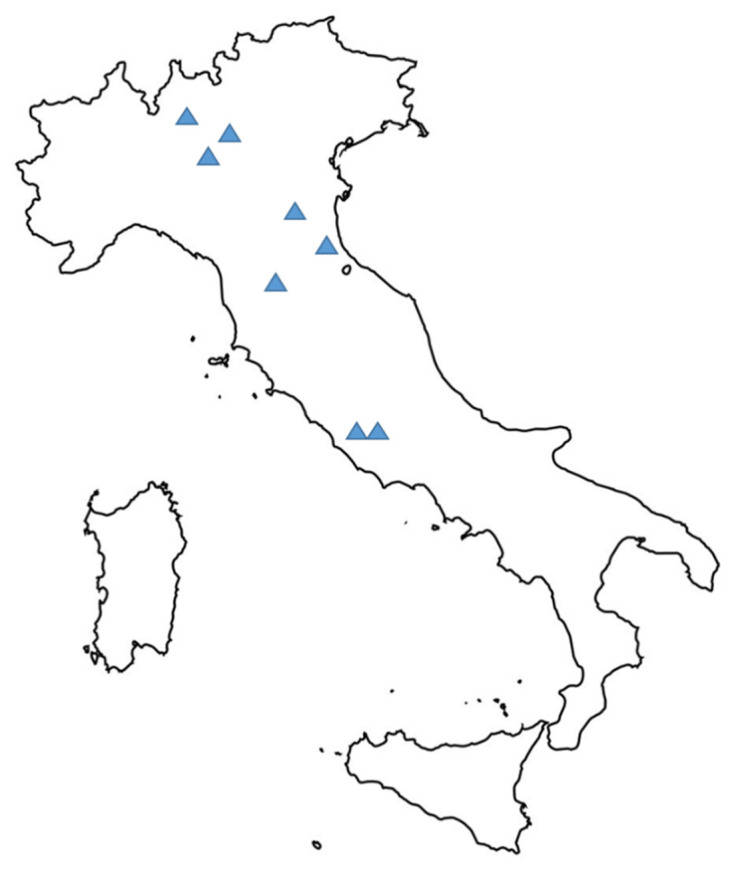
Geographical distribution of participating centers. The detailed list of the eight participating centers is available as supplementary material (Table S1).

We conducted an observational, retrospective, national multicenter, case-control study with 1:3 matching to assess the incidence, clinical characteristics and outcomes of COVID-19 patients with CDI. In addition, we evaluated risk factors associated with the occurrence of CDI in COVID-19 patients. The study was performed in 8 acute-care Italian hospitals admitting COVID-19 patients, between February 2020 and July 2020 (Figure 1 and Table S1). All the hospitals have an Infectious Disease Unit. The study was approved by the Ethics Committees of the participant hospitals.

### 2.1. Study Design

Hospitalized adult (>18 years old) patients with COVID-19 and CDI were identified from the databases of the participant centers. Cases were defined as COVID-19 patients with CDI; controls were COVID-19 patients without CDI. Cases were matched 1:3 with controls. Demographic, epidemiological and clinical data (COVID-19 onset and clinical characteristics, medications given for COVID-19, antimicrobial treatments before and after the diagnosis of COVID-19, laboratory data, CDI onset and characteristic, and patient’s outcome) were collected in clinical record forms (CRF) (Table S2).

Controls were matched to cases according to the following criteria:

1. Same gender
2. Hospitalization in the same hospital and in the same unit
3. Same date of hospital admission ±7 days
4. Same age ±3 years

All cases and controls were followed up to 30 days from their hospital discharge to assess for new onset of diarrhoea, recurrence of CDI, and mortality at 30 days from the hospital discharge.

The definitions of CDI, microbiological evidence of C. difficile, CDI recurrence, mild CDI, severe CDI and complicated CDI and the definitions of the clinical syndromes associated with COVID-19 adopted in the study are described in Table S3.

### 2.2. Data Analysis

The incidence of CDI among all COVID-19 patients admitted to the participating hospitals was calculated using as numerator the number of CDI cases and as denominator the number of days of hospitalization of the COVID-19 patients (× 10,000). The characteristics of the study population and the patient outcome were evaluated by means of descriptive statistics. The potential correlations between CDI and clinical variables of COVID-19 (infection onset, severity) and laboratory findings were analysed by univariate and multivariate analysis. To identify risk factors for onset of CDI in COVID-19 patients and any determinants of delayed diagnosis of CDI, the characteristics of the CDI group were compared to the control group by means of univariate and multivariate analysis.

### 2.3. Statistical Analysis

Quantitative variables were tested for normal distribution and compared by means of a paired t-test. Qualitative differences between groups were assessed by use of Fisher’s exact test. The precision of odd ratio (OR) was determined by calculating a 95% confidence interval (CI). A *p* value less than 0.05 was considered statistically significant. Variables from the univariate analysis were considered for inclusion in multivariate logistic regression analysis if *p*-value was less than 0.05. Backward stepwise logistic regression was performed, and the model that was considered biologically plausible and had the lowest −2 log-likelihood ratio was chosen as the final model. Statistical analysis was performed using the software program IBM SPSS version 24.

## 3. Results

### 3.1. CDI Incidence among COVID-19 Patients

Overall, during the study period, a total of 40,315 patients were admitted to the eight participant hospitals; of these, 8402 were COVID-19 patients. The mean hospital stay for COVID-19 patients was 13.8 days (range 1–59 days). Thirty-eight CDI cases were identified, including 32 hospital-onset CDI (HO-CDI) and 6 community-onset, healthcare-associated CDI (CO-HCA-CDI) cases. Therefore, during the study period 32 COVID-19 patients developed HO-CDI, corresponding to an HO-CDI prevalence of 0.38%, and an HO-CDI incidence of 4.4 × 10,000 patient days ranging in the hospitals from 0.7 to 12.3 × 10,000 patient days (Table S4).

### 3.2. Clinical Features of Clostridioides Difficile Infection in COVID-19 Patients

The demographic and epidemiological data, the comorbidities, the clinical characteristics and the outcome of the 38 COVID-19 patients with CDI and of the 114 controls included in the study are described in Table 1. The mean laboratory findings at the admission of the 38 COVID-19 patients with CDI and of the 114 controls are shown in Table 2. The CDI characteristics, severity, management and the follow-up of the 38 COVID-19 patients with CDI included in the study are shown in Table 3.

Among the 38 COVID-19 patients with CDI, 23 (60.5%) patients were female (Table 1). The mean age of the 38 COVID-19 patients with CDI was 79 years, ranging between 53 and 97 years. The mean age-adjusted Charlson co-morbidity index (CCI) at admission was 6.6. Elevated inflammatory markers and abnormal blood chemistries were common among COVID-19 patients with CDI, but the mean albumin value was the only difference compared to COVID-19 controls (Table 2).

Among the 38 COVID-19 patients with CDI included in the study, 32/38 (84.2%) were diagnosed with HO-CDI and 6/38 (15.8%) with CO-HCA-CDI (Table 3). Thirty-six out of 38 (94.7%) CDI patients had a primary CDI, and 2 (5.3%) a recurrence. In 32 out of 38 (84.2%), the diarrhoea onset and the CDI diagnosis occurred after the COVID-19 diagnosis. Regarding CDI severity, 23 (60.5%), 11 (28.9%) and 4 (10.5%) had mild, severe and complicated CDI, respectively. Overall, the mean length of the in-hospital stay was 35 days, ranging between 1 and 96 days (Table 1). Regarding risk factors for CDI before the admission 21/38 (55.2%), 25/38 (65.7%) and 8/38 (21%) CDI patients received antibiotics, proton pump inhibitors (PPI) and steroids (dexamethasone or methylprednisolone) in the previous two months, respectively (Table 1).

Table 1 shows also patient comorbidities. At admission five (13.1%), patients had a concomitant bacterial infection other than CDI, including two patients with bacterial pneumonia, one patient with acute cholecystitis, one patient with *S. aureus* endocarditis and one *K. pneumoniae* bacteraemia.

Regarding COVID-19 severity during the hospitalisation, 7 (18.4%), 15 (39.4%), 12 (31.5%), 3 (7.8%) and 1 (2.6%) had asymptomatic, mild pneumonia, severe pneumonia, ARDS and septic shock, respectively. As of medications administered for COVID-19 during the hospital stay, 35 (92.1%), 25 (65.7%), 26 (68.4%), 14 (36.8%) and 9 (23.6%) patients received low molecular weight heparin, PPI, chloroquine, lopinavir or darunavir and steroids, respectively (Table 1). Additionally, 32/38 (84.2%) CDI patients were treated with broad-spectrum antimicrobials (Table 1). The most common antimicrobial class was beta-lactam, which was administered in 21 (55.2%) patients. Macrolides (specifically azithromycin), carbapenems, glycopeptides and quinolones were administered in 12 (31.5%), 11 (28.9%), 5 (13.1%), 4 (10.5%) patients, respectively. Of note, during the hospital stay 18/38 (47.3%) CDI patients developed a concomitant bacterial infection (Table 3), including nine urinary tract infections, five sepsis, two abdominal infections and two bacterial pneumonia.

About outcomes, 19/38 (50%) recovered and were discharged without complications; 8/38 (21.1%) developed complications at discharge, including seven patients with muscle weakness and pressure ulcers and one patient with decompensated chronic heart failure and orthostatic hypotension. Eleven out of 38 (28.9%) patients died in the hospital. CDI was the main cause of death in one of these patients, while septic shock, respiratory failure and heart failure were considered the main cause of death in seven, two and one patients, respectively.

Therefore, 27/38 (71.0%) COVID-19 patients with CDI survived and were discharged at home. Of them, 21 patients were followed up to 30 days from the hospital discharge; no data are available for the remaining six patients. Of these 21 patients, 15 (71.4%) fully recovered at home, presenting no subsequent recurrent CDI (rCDI) episode. Three patients (14.3%) developed rCDI and were readmitted in hospital; one of them died due to rCDI. Three other patients (14.3%) were readmitted in the hospital for reasons other than CDI, and one died during the hospital stay.

### 3.3. Risk Factors for the Onset of CDI in COVID-19 Patients

Table 4 shows the results of the logistic regression analysis to identify the factors associated with the likelihood of CDI during COVID-19 infection (Table 4).

Cases and controls were different for previous hospitalisations in the two months before the current admission; i.e., 65.7% versus 22.8%, respectively (*p* = 0.0003) (Table 1). Additionally, the proportion of cases who received broad-spectrum antibiotics during the hospital stay was higher than among controls (84.2% versus 55.2%, *p* = 0.001).

Logistic regression analysis identified the hospitalisation in the previous two months [OR: 5.5 (95% CI: 2.2–13.8), *p* = 0.001], the administration of steroids in the previous two months [OR: 8.4 (95% CI: 1.7–40.8), *p* = 0.008] and the administration of antibiotics during the hospital stay [OR: 5.6 (95% CI: 1.7–18.4), *p* = 0.004] as independent risk factors associated with CDI in COVID-19 patients (Table 4).

## 4. Discussion

During this COVID-19 pandemic, most hospitals experienced overcrowding, emergency situations, and the need for strict isolation measures for infected patients, which in turn represented major factors for decreasing attention toward infection control measures. Paradoxically, in the management of SARS-CoV-2, a viral infection, there was an overuse of antibiotics, with little concern for established antimicrobial stewardship programs [13,14]. These conditions have a potential impact on both antimicrobial resistance and CDI. In this study, we observed an HO-CDI rate among COVID-19 patients of 4.4 per 10,000 patient days; this CDI incidence is within the range of incidence observed in previous reports performed before the COVID-19 pandemic in Italy and in Europe [15,16,17]. We observed wide differences in CDI incidence among the participating centers, from 0.7 to 12.3 per 10,000 patient days with rates higher than 10 per 10,000 patients days in two centers where a cluster of cases, i.e., 4/11 in center 2 and 2/2 in center 7, occurred in one week during the study period. All the centers had guidelines on infection prevention and control measures and on antimicrobial stewardship; however, as a limitation, no information was available on antibiotic usage or breaks in infection prevention and control measures at the centers with higher CDI incidence rates. While the overall rate of CDI did not appear to have increased during the pandemic, the full impact of COVID-19 on CDI may not yet have been realized considering the delayed effect of other seasonal respiratory viral infections on CDI rates [18]. Interestingly, our study reported a worse outcome for CDI COVID-19 patients in comparison with COVID-19 patients without CDI. Indeed, the percentage of CDI COVID-19 patients recovering without complications at discharge was significantly lower than the control patients, and had longer in-hospital stays than control patients. Our study identified also that healthcare exposures and usage of antibiotics during the in-hospital stay were independently associated with the risk for CDI among COVID-19 patients. These findings confirm that even during the COVID-19, frail patients with previous healthcare exposure are at higher risk to develop CDI, and that antimicrobial exposure is a strong risk factor for developing CDI. These findings also confirm that infection prevention and control is a crucial strategy to reduce the CDI rate during the COVID-19 pandemic. Indeed, in our study, an extremely high percentage of COVID-19 patients received broad-spectrum antibiotics during their hospital stay, even though there is no evidence of any benefit of empirical antimicrobial administration for pneumonia during SARS-COV-2 infection. As a consequence, COVID-19 patients unnecessarily exposed to antibiotics are placed at risk for serious adverse events, including CDI, with no clinical benefit.

Our study highlighted some of the risk factors for CDI in COVID-19 patients, but there are likely other ones that we were not able to identify. Indeed, SARS-CoV-2 infection may alter the onset and the clinical course of CDI patients through different mechanisms, including:

(1) Derangement of the innate and the adaptive immune response due to the SARS-CoV-2 virus replication [19];
(2) Damage to the host gastrointestinal barrier exerted by the SARS-CoV-2 virus [20,21];
(3) Detrimental effect to the gut microbiome exerted by SARS-CoV-2 infection and/or hospitalization and antimicrobials administration during the treatment of COVID-19 patients [22];
(4) Delayed CDI diagnosis due to the misleading interpretation of gastrointestinal symptoms in COVID-19 patients as caused by the action of SARS-CoV-2 virus instead of Clostridioides difficile [23,24];
(5) Reduced adherence to infection prevention and control measures during the management of COVID-19 patients [13,14].

Future studies are needed to clarify the interaction mechanisms between COVID-19 and CDI.

In conclusion, the results of our study underline the importance of infection prevention and control and of rational use of antibiotics, in the management of COVID-19 patients. In our opinion, antimicrobial stewardship should be incorporated into guidelines and recommendations for the optimal management of COVID-19 patients.

## Figures and Tables

**Table 1:** Demographic and epidemiological data, comorbidities, clinical characteristics of the Coronavirus Disease 2019 (COVID-19) and outcome of the 38 COVID-19 patients with CDI and of the 114 COVID-19 controls included in the study. CCI: Charlson Co-morbidity Index. LTHCF: long-term health care facility. ARDS: Acute Respiratory Distress Syndrome. LMWH: Low Molecular Weight Heparin. CI: confidence interval.

	CDI Patients (*n*: 38)	Control COVID-19 Patients (*n*: 114)	Fisher’s Test (Paired t Test for CCI)	Odds Ratio (95% CI)
Female gender	23 (60.5%)	69 (60.5%)		
Age (years)	79 (range: 53–97)	79 (range: 51–96)		
Comorbidities				
No comorbidities	1 (2.6%)	10 (8.7%)	*p*: 0.2	
Cardiovascular disease	32 (84.2%)	65 (57.0%)	*p*: 0.009	3.2 (1.2–8.3)
Heart failure	8 (21.0%)	20 (17.5%)	*p*: 0.4	
Diabetes	7 (18.4%)	27 (23.6%)	*p*: 0.3	
Renal failure	12 (31.5%)	20 (17.5%)	*p*: 0.2	
Dialisis	1 (2.6%)	3 (2.6%)	*p*: 0.4	
Chronic liver failure	2 (5.2%)	4 (4.2%)	*p*: 0.6	
Neurological disease	16 (42.1%)	38 (33.3%)	*p*: 0.4	
Vasculitis	2 (5.2%)	0	*p*: 0.07	
COPD	10 (26.3%)	20 (17.5%)	*p*: 0.3	
Solid Cancer	8 (21.0%)	24 (21.0%)	*p*: 0.5	
Blood cancer	3 (7.8%)	1 (0.9%)	*p*: 0.06	
Transplant, immunodeficiency, immunosuppression	3 (7.8%)	0	*p*: 0.015	1.1 (1–1.2)
Other concomitant infections at admission	5 (13.1%)	2 (1.7%)	*p*: 0.011	8.4 (1.5–45)
Mean age-adjusted CCI at admission	6.6	5.6	*p*: 0.03	-
Hospitalisation in the previous two months	25 (65.7%)	26 (22.8%)	*p*: 0.0003	6.1 (2.7–13.7)
Transferred to the hospital from a LTHCF	16 (42.1%)	36 (31.5%)	*p*: 0.3	
Antibiotic in the previous two months	21 (55.2%)	21 (18.4%)	*p*: 0.0001	4.3 (2–9.5)
Proton pump inhibitors in the previous two months	25 (65.7%)	42 (36.8%)	*p*: 0.004	2.9 (1.3–6.3)
Steroids in the previous two months °	8 (21.0%)	3 (2.6%)	*p*: 0.002	7.2 (2–25.7)
COVID-19 severity				
Asymptomatic	7 (18.4%)	24 (21.0%)	*p*: 0.5	
Mild pneumonia	15 (39.4%)	32 (28.0%)	*p*: 0.2	
Severe pneumonia	12 (31.5%)	33 (28.9%)	*p*: 0.3	
ARDS	3 (7.8%)	24 (21.0%)	*p*: 0.05	0.3 (0.1–1.1)
Sepsis	1 (2.6%)	1 (0.8%)	*p*: 0.4	
Intensive care unit stay during the hospitalisation	5 (13.1%)	13 (11.4%)	*p*: 0.5	
Complications during hospitalization				
Acute pulmonary embolism	2 (5.2%)	4 (3.5%)	*p*: 0.5	
Respiratory failure	16 (42.1%)	42 (36.8%)	*p*: 0.4	
Surgery	1 (2.6%)	0	*p*: 0.4	
Bacterial superinfections	18 (47.3%)	16 (14.0%)	*p*: 0.0007	5.9 (2.5–13.7)
Medication for COVID-19 during the hospital stay				
Lopinavir or darunavir	14 (36.8%)	44 (38.5%)	*p*: 0.5	
Chloroquine	26 (68.4%)	80 (70.1%)	*p*: 0.4	
Remdesivir	0	1 (0.8%)	*p*: 0.7	
Biologics	3 (7.8%)	20 (17.5%)	*p*: 0.1	
LMWH	35 (92.1%)	87 (76.3%)	*p*: 0.1	
Steroids	9 (23.6%)	42 (36.8%)	*p*: 0.07	
Proton pump inhibitors	25 (65.7%)	72 (63.1%)	*p*: 0.2	
Antibiotics	32 (84.2%)	63 (55.2%)	*p*: 0.001	4.1 (1.6–10.7)
Patients outcome				
Recovered without complications *	19 (50%)	74 (64.9%)	*p*: 0.01	0.4 (0.1–0.8)
Recovered with complications *	8 (21.0%)	8 (7.0%)	*p*: 0.1	
Deceased	11 (28.9%)	25 (21.9%)	*p*: 0.1	
Total length of in-hospital stay (days)	35 (range: 1–96)	19.4 (range: 1–88)	*p*: 0.0007	

* at discharge, including muscle weakness, pressure ulcers, chronic heart decompensation. ° dexamethasone or methylprednisolone.

**Table 2:** Mean laboratory findings at the admission of the 38 COVID-19 patients with CDI cases and of the 114 controls included in the study. Ns: not significant.

	CDI Patients (*n*: 38)	Control COVID-19 Patients (*n*: 114)	Paired t-Test (*p*-Value)
C Reactive Protein (mg/dL)	16.9 ± 22.2	17.1 ± 24.3	0.1
Ferritin (ng/dL)	1247.0 ± 3194.7	572.1 ± 494.4	0.09
Fibrinogen (mg/dL)	546.1 ± 194.8	464.1 ± 172.8	0.06
D-dimer (ng/mL)	3735.3 ± 4945.0	2527.0 ± 3931,0	0.2
Interleukin-6 (pg/mL)	25.5 ± 37.3	29.3 ± 47.8	0.8
Total white blood cells peripheral count (10^3^ cells/µL)	12.160 ± 9.780	8.149 ± 4.863	0.05
Neutrophils peripheral count (10^3^ cells/µL)	9.950 ± 9.626	6.245 ± 4.562	0.05
Blood creatinine value (mg/dL)	2.29 ± 5.56	1.35 ± 1.34	0.1
Blood albumin value (g/dL)	2.74 ± 0.54	3.07 ± 0.51	0.02

**Table 3:** *Clostridioides difficile* infection characteristics, severity, management, outcome and follow-up of the 38 COVID-19 patients with CDI included in the study. CDI: *Clostridioides difficile* infection. rCDI: recurrent CDI episode.

	COVID-19 Patients with CDI (*n*: 38)
Hospital-onset CDI	32 (84.2%)
Community-onset, healthcare-associated CDI	6 (15.8%)
First CDI episode	36 (94.7%)
Recurrence of CDI	2 (5.3%)
Diarrhea onset before the COVID-19 diagnosis	6 (15.8%)
Diarrhea onset after the COVID-19 diagnosis	32 (84.2%)
CDI severity at diagnosis	
Mild	23 (60.5%)
Severe	11 (28.9%)
Complicated	4 (10.5%)
Administered anti-CD antimicrobial treatment	
Vancomycin	26 (68.4%)
Vancomycin and Metronidazole	5 (13.1%)
Metronidazole	3 (7.8%)
Vancomycin and Fidaxomicin	3 (7.8%)
Fidaxomicin	1 (2.6%)
Surgery for complicated CDI	0
Stool transplantation	2 (5.2%)
Follow up at 30 days from the discharge	
Deceased before the discharge	11 (28.9%)
Follow up after discharge not available	6 (15.7%)
Patients followed-up at 30 days from the discharge	21
Recovered at home, no subsequent rCDI	15 (71.4%)
Readmission in hospital for subsequent rCDI	3 (14.3%)
Readmission in hospital, no subsequent rCDI	3 (14.3%)
Deceased, CDI-related	1 (4.7%)
Deceased, not CDI-related	1 (4.7%)

**Table 4:** Factors associated with the likelihood of CDI during COVID-19 infection. Logistic regression analysis. CI: confidence interval.

	CDI Patients (*n*: 38)	Control COVID-19 Patients (*n*: 114)	Univariate Analysis		Multivariate Analysis	
			*p*-Value	Odds Ratio (95% CI)	*p*-Value	Odds Ratio (95% CI)
Comorbidities						
Cardiovascular disease	32 (84.2%)	65 (57.0%)	0.009	3.2 (1.2–8.3)	0.1	
Transplant, immunodeficiency, immunosuppression	3 (7.8%)	0	0.015	1.1 (1–1.2)	0.9	
Hospitalisation in the previous two months	25 (65.7%)	26 (22.8%)	0.0003	6.1 (2.7–13.7)	0.001	5.5 (2.2–13.8)
Antibiotic in the previous two months	21 (55.2%)	21 (18.4%)	0.0001	4.3 (2–9.5)	0.4	
Proton pump inhibitors in the previous two months	25 (65.7%)	42 (36.8%)	0.004	2.9 (1.3–6.3)	0.07	
Steroids in the previous two months °	8 (21.0%)	3 (2.6%)	0.002	7.2 (2–25.7)	0.008	8.4 (1.7–40.8)
Medication for COVID-19 during the hospital stay						
Antibiotics	32 (84.2%)	63 (55.2%)	0.001	4.1 (1.6–10.7)	0.004	5.6 (1.7–18.4)

° dexamethasone or methylprednisolone.

## Supplementary Materials

The following are available online at https://www.mdpi.com/2077-0383/9/12/3855/s1, Table S1: List of participating centres, Table S2: Data collected from the clinical records of the patients enrolled in the study, either cases or controls. Table S3: The definitions of CDI, microbiological evidence of CDI, CDI recurrence, mild CDI, severe CDI and complicated CDI and the definitions of the clinical syndromes associated with COVID-19 adopted in the study. Table S4: Hospital-onset CDI incidence among the COVID-19 patients admitted in the 8 participant Infectious Diseases Units during the study period. References [25,26] are cite d in the supplementary material.

## References

[1] Olsen S.J., Chen M.-Y., Liu Y.-L., Witschi M., Ardoin A., Calba C., Mathieu P., Masserey V., Maraglino F., Marro S. (2020). Early Introduction of Severe Acute Respiratory Syndrome Coronavirus 2 into Europe. Emerg. Infect. Dis..

[2] Onder G., Rezza G., Brusaferro S. (2020). Case-Fatality Rate and Characteristics of Patients Dying in Relation to COVID-19 in Italy. JAMA.

[3] WHO Coronavirus Disease (COVID-19). https://www.who.int/docs/default-source/coronaviruse/situation-reports/20200921-weekly-epi-update-6.pdf?sfvrsn=d9cf9496_6.

[4] Huttner B.D., Catho G., Pano-Pardo J.R., Pulcini C., Schouten J. (2020). COVID-19: Don’t neglect antimicrobial stewardship principles. Clin. Microbiol. Infect..

[5] Huang C., Wang Y., Li X. (2020). Clinical features of patients infected with 2019 novel coronavirus in Wuhan, China. Lancet.

[6] Chen N., Zhou M., Dong X. (2020). Epidemiological and clinical characteristics of 99 cases of 2019 novel coronavirus pneumonia in Wuhan, China: A descriptive study. Lancet.

[7] Chen T., Wu D., Chen H., Yan W., Yang D., Chen G., Ma K., Xu D., Yu H., Wang H. (2020). Clinical characteristics of 113 deceased patients with coronavirus disease 2019: Retrospective study. BMJ.

[8] Yang X., Yu Y., Xu J., Shu H., Xia J., Liu H., Wu Y., Zhang L., Yu Z., Fang M. (2020). Clinical course and outcomes of critically ill patients with SARS-CoV-2 pneumonia in Wuhan, China: A single-centered, retrospective, observational study. Lancet Respir. Med..

[9] Beović B., Doušak M., Ferreira-Coimbra J., Nadrah K., Rubulotta F., Belliato M., Berger-Estilita J., Ayoade F., Rello J., Erdem H. (2020). Antibiotic use in patients with COVID-19: A ‘snapshot’ Infectious Diseases International Research Initiative (ID-IRI) survey. J. Antimicrob. Chemother..

[10] Sandhu A., Tillotson G., Polistico J., Salimnia H., Cranis M., Moshos J., Cullen L., Jabbo L., Diebel L., Chopra T. (2020). Clostridioides difficile in COVID-19 Patients, Detroit, Michigan, USA, March–April 2020. Emerg. Infect. Dis..

[11] Páramo-Zunzunegui J., Ortega-Fernández I., Calvo-Espino P., Diego-Hernández C., Ariza-Ibarra I., Otazu-Canals L., Danés-Grases J., Menchero-Sánchez A. (2020). Severe Clostridium difficile colitis as potential late complication associated with COVID-19. Ann. R. Coll. Surg. Engl..

[12] Cataldo M.A., Granata G., Petrosillo N. (2017). Clostridium difficile infection: New approaches to prevention, non-antimicrobial treatment, and stewardship. Expert. Rev. Anti Infect. Ther..

[13] Rawson T.M., Moore L.S., Castro-Sánchez E., Charani E., Davies F., Satta G., Ellington M.J., Holmes A.H. (2020). COVID-19 and the potential long-term impact on antimicrobial resistance. J. Antimicrob. Chemother..

[14] Spernovasilis N.A., Kofteridis D.P. (2020). COVID-19 and antimicrobial stewardship: What is the interplay?. Infect. Control Hosp. Epidemiol..

[15] Di Bella S., Musso M., Cataldo M.A., Meledandri M., Bordi E., Capozzi D., Cava M.C., Chiaradonna P., Prignano G., Petrosillo N. (2013). Clostridium difficile infection in Italian urban hospitals: Data from 2006 through 2011. BMC Infect. Dis..

[16] Cioni G., Viale P., Frasson S., Cipollini F., Menichetti F., Petrosillo N., Brunati S., Spigaglia P., Vismara C., Bielli A. (2016). Epidemiology and outcome of Clostridium difficile infections in patients hospitalized in Internal Medicine: Findings from the nationwide FADOI-PRACTICE study. BMC Infect. Dis..

[17] Davies K.E., Davis G., Barbut F., Eckert C., Petrosillo N., Pisapia R., Gärtner B., Berger F.K., Reigadas-Ramirez E., Bouza E. (2020). Factors affecting reported Clostridioides difficile infection rates; the more you look the more you find, but should you believe what you see?. Anaerobe.

[18] Gilca R., Fortin E., Frenette C., Longtin Y., Gourdeau M. (2012). Seasonal variations in Clostridium difficile infections are associated with influenza and respiratory syncytial virus activity independently of antibiotic prescriptions: A time series analysis in Quebec, Canada. Antimicrob. Agents Chemother..

[19] Blanco-Melo D., Nilsson-Payant B.E., Liu W.-C., Uhl S., Hoagland D., Møller R., Jordan T.X., Oishi K., Panis M., Sachs D. (2020). Imbalanced Host Response to SARS-CoV-2 Drives Development of COVID-19. Cell.

[20] Cha M.H., Regueiro M., Sandhu D.S. (2020). Gastrointestinal and hepatic manifestations of COVID-19: A comprehensive review. World. J. Gastroenterol..

[21] Sultan S., Altayar O., Siddique S.M., Davitkov P., Feuerstein J.D., Lim J.K., Falck-Ytter Y., El-Serag H.B. (2020). AGA Institute Rapid Review of the Gastrointestinal and Liver Manifestations of COVID-19, Meta-Analysis of International Data, and Recommendations for the Consultative Management of Patients with COVID-19. Gastroenterology.

[22] Zuo T., Zhang F., Lui G.C., Yeoh Y.K., Li A.Y., Zhan H., Wan Y., Chung A.C., Cheung C.P., Chen N. (2020). Alterations in Gut Microbiota of Patients With COVID-19 During Time of Hospitalization. Gastroenterology.

[23] Lakkasani S., Chan K., Shaaban H.S. (2020). Clostridiodes difficile in COVID-19 Patients, Detroit, Michigan, USA, March–April 2020. Emerg. Infect. Dis..

[24] Han C., Duan C., Zhang S., Spiegel B., Shi H., Wang W., Zhang L., Lin R., Liu J., Ding Z. (2020). Digestive Symptoms in COVID-19 Patients With Mild Disease Severity. Am. J. Gastroenterol..

[25] Debast S.B., Bauer M.P., Kuijper E.J. (2014). European Society of Clinical Microbiology and Infectious Diseases: Update of the treatment guidance document for Clostridium difficile infection. European Society of Clinical Microbiology and Infectious Diseases. Clin. Microbiol. Infect..

[26] Crobach M., Planche T., Eckert C., Barbut F., Terveer E.M., Dekkers O., Wilcox M., Kuijper E.J. (2016). European Society of Clinical Microbiology and Infectious Diseases: Update of the diagnostic guidance document for Clostridium difficile infection. Clin. Microbiol. Infect..

