# Supplementary material for: The Burden of Clostridioides Difficile Infection during the COVID-19 Pandemic: A Retrospective Case-Control Study in Italian Hospitals (CloVid)
Source: J Clin Med. 2020 Nov 27;9(12):3855. doi: 10.3390/jcm9123855 (PMC7761275; doi:10.3390/jcm9123855)
Supplement: Supplementary file 1 [file jcm-09-03855-s001.pdf]

Journal of
Clinical Medicine

MDPI

Table S1. List of participating centres.

List of participating centres
IRCCS Istituto Nazionale Malattie Infettive "L. Spallanzani", Rome (co-ordinating centre)
Fondazione Policlinico Universitario Agostino Gemelli IRCCS, Rome
ASST Grande Ospedale Metropolitano Niguarda, Milan
Ospedale di Piacenza "Guglielmo da Saliceto", Piacenza
Ospedale di Cremona, Cremona
Policlinico Sant'Orsola-Malpighi, Bologna
Azienda Ospedaliero-Universitaria Careggi, Florence
Unità Operativa Complessa di Malattie Infettive, Rimini, Forlì and Cesena

Table S2. Data collected from the clinical records of the patients enrolled in the study, either cases or controls.

**Data collected from the clinical records of the enrolled patients, either cases or controls**
Gender, age
Date of admission in the participant hospital
Date of COVID-19 onset and date of COVID-19 diagnosis
Intensive Care stay during the hospitalization
Patient' comorbidities, including cardiovascular disease, heart failure, diabetes, renal failure, dialisis, chronic liver failure, neurological disease, vasculitis, COPD, solid or blood cancer, transplant, immunodeficiency, immunosuppression, other concomitant infections
Previous hospitalization, previous administration of a broad-spectrum antibiotic, proton pump inhibitors, steroids
Severity of the COVID-19 during the hospitalization
Complications occurred during the hospitalization, including acute pulmonary embolism, respiratory failure, surgery, other coinfections or superinfections
Medication received for COVID-19
Laboratory findings at admission, including the values of C reactive protein, ferritin, fibrinogen, d-dimer, interleukin-6, white blood cells, neutrophils, creatininemia, albuminemia.
Patient outcome
**Data collected from the clinical records of the enrolled cases, with CDI and COVID-19**
Date of diarrhea onset
CDI severity
Characteristic of the CDI (first CDI episode, recurrent CDI)
Medications received for the CDI treatment, including dosages and administration route of vancomycin, metronidazole, fidaxomicin, bezlotoxumab, stool transplantation, surgery for complicated CDI

Table S3. The definitions of CDI, microbiological evidence of CDI, CDI recurrence, mild CDI, severe CDI and complicated CDI and the definitions of the clinical syndromes associated with COVID-19 adopted in the study. HO-CDI: hospital-onset CDI; CO-HCA-CDI: community-onset healthcare associated CDI.

**CDI**: A clinical picture characterized by diarrhea or ileum or toxic megacolon in the presence of microbiological evidence of CDI [25,26].
**CO-HCA-CDI** when symptom onset was <48 hours from the admission in patients who had been in contact with a health care facility (health care worker status, admission to hospital, health care procedures) during the 3 months before the symptom onset;

**HO-CDI** when symptom onset was >48 hours from hospital admission.
**Microbiological evidence of CDI**:
o GDH positive antigen + A/B positive toxins (enzyme immunoassay) or
o GDH positive antigen, A/B negative toxins (immunoenzymatic method) + positivity of the amplification of the genes that encode C. difficile toxins or
o Positivity of the amplification of the genes that encode C. difficile toxins + A/B positive toxins by enzyme immunoassay
**Recurrence of CDI**: The onset of a new episode of CDI after at least two days from the resolution of the diarrhea and after the end of the antimicrobial treatment of the previous CDI episode [25,26].
**Mild CDI**: CDI in the absence of the following criteria: fever (> 38.5°), chills, hemodynamic instability, signs of ileus or peritonitis, leukocytosis (leukocytes >15,000 cells/μl) creatininemia increase >1.5 times the values before the infection, increase in serum lactates, histological evidence of pseudo-membranous colitis, radiological evidence of ileus or ascites [25,26].
**Severe CDI**: CDI in the presence of at least one of the following criteria: fever (> 38.5°), chills, hemodynamic instability, signs of ileus or peritonitis, leukocytosis (leukocytes >15,000 cells/μl) creatininemia increase >1.5 times the values before the infection, increase in serum lactates, histological evidence of pseudo-membranous colitis, radiological evidence of ileus or ascites [25,26].
**Complicated CDI**: An episode of CDI complicated by toxic megacolon, intensive care unit hospitalization, sepsis, surgery or death caused by CDI.
**Clinical syndromes associated with COVID-19, WHO criteria:**
**Uncomplicated disease** - Patients with uncomplicated upper respiratory tract viral infection. Patients may have non-specific symptoms such as fever, malaise, cough, pharyngodynia, nasal congestion, headache or muscle pain. These patients have no signs of dyspnoea, dehydration or sepsis. No evidence of viral pneumonia or hypoxia.
**Mild pneumonia** - Patient with clinical signs of pneumonia (fever, cough, dyspnoea, fast breathing) but no signs of severe pneumonia, including SpO_2 ≥90% on room air.

Table S4. Hospital-onset CDI incidence among the COVID-19 patients admitted in the 8 participant Infectious Diseases Units during the study period.

Participant Infectious Disease Units	Number of admitted COVID-19 cases	Mean hospital stay for COVID-19 cases	Number of hospital-onset CDI cases among COVID-19 patients	Hospital-onset CDI incidence among COVID-19 patients (per 10,000 patient days)
#1	646	18	4	3,4
#2	789	13,4	11	10,4
#3	901	17,3	2	1,2
#4	1760	11,8	5	2,4
#5	2187	13,1	2	0,7
#6	1097	16,9	3	1,6
#7	178	9,1	2	12,3
#8	844	10,6	3	3,3
Total	8402	13,8	32	4,4

© 2020 by the authors. Licensee MDPI, Basel, Switzerland. This article is an open access article distributed under the terms and conditions of the Creative Commons Attribution (CC BY) license (http://creativecommons.org/licenses/by/4.0/).
